# Geometric Effect of the Photo Responsivity of Organic Phototransistors

**DOI:** 10.3390/ma18143349

**Published:** 2025-07-17

**Authors:** Chengtai Li, Xiaochen Ren

**Affiliations:** Key Laboratory of Organic Integrated Circuits, Ministry of Education & Tianjin Key Laboratory of Molecular Optoelectronic Sciences, Department of Chemistry, School of Science, Tianjin University Collaborative Innovation Center of Chemical Science and Engineering (Tianjin), Tianjin 300072, China; lichengtai@tju.edu.cn

**Keywords:** responsivity, organic phototransistor, organic field-effect transistor, geometric effect

## Abstract

Organic phototransistors exhibit considerably higher photoresponsivity than diode-like photodetectors owing to gate-field-effect amplification. However, the conventional definition of photoresponsivity (*R*) fails to accurately capture the photoresponsivity trends of transistor-based photodetectors. This study systematically investigates the impact of device geometry—specifically the width-to-length (*W*/*L*) ratio and photosensitive area—on the responsivity and photocurrent of organic phototransistors. The experimental results reveal that increasing the *W*/*L* ratio or decreasing the device area substantially enhances responsivity. A detailed analysis based on the definition of responsivity is presented herein. Finally, we introduce a channel-width-normalized responsivity to compensate for geometric effects, enabling a more accurate evaluation of device performance across different device structures. Overall, our results indicate the potential for optimizing organic phototransistors by tuning their geometric parameters.

## 1. Introduction

The widespread adoption of organic photodetectors, specifically organic phototransistors (OPTs) based on organic field-effect transistors (OFETs), has prompted continuous research into strategies for optimizing their performance, particularly in terms of photoresponsivity and sensitivity. As an important class of organic semiconductor devices, OFETs offer advantages such as mechanical flexibility, low cost, and the potential for large-area fabrication. These properties make them well-suited for applications in flexible displays, sensors, and photodetectors [1,2]. Despite these advantages, however, enhancing the photoresponsivity of OFETs remains a major challenge in improving overall device performance [3,4].

Photoresponsivity (*R*) is a key performance indicator of photodetectors, defined as the ratio of the generated photocurrent (*I*_ph_) to the incident optical power (*P*_opt_) [5]. To date, most studies have focused on enhancing the responsivity of OFETs by modifying the properties of their organic materials, such as by improving charge transport or optimizing molecular alignment, as well as by introducing structural modifications to facilitate charge injection and transport [6,7]. However, the influence of device geometry, particularly the width-to-length ratio (*W*/*L*), on responsivity has received comparatively less attention [8].

This is critical because the photocurrent flowing through OFETs is influenced not only by the incident optical power and the material properties of the detector but also by the geometrical structure of the device, particularly the ratio of its channel width (*W*) to its channel length (*L*). Theoretically, increasing the *W*/*L* ratio generates a higher photocurrent, which, in turn, influences *R*, as a larger *W*/*L* ratio amplifies the current response [9,10]. However, the geometric influence on responsivity in phototransistors has generally been overlooked in existing research [11]. Under these circumstances, acquiring a systematic understanding of geometric effects on the photoresponsivity of OPTs is critical.

In this study, we propose a new framework for correcting responsivity by systematically examining the influence of the *W*/*L* ratio and device area on the responsivity of OFETs, as well as exploring strategies to enhance photodetector performance through geometric parameter optimization. First, we review the fundamental concept of responsivity, following which we examine the impact of device area and geometry on photocurrent. Finally, we validate the effect of *W*/*L* ratio optimization on device responsivity using experimental data. Based on these analyses, we aim to provide new insights for optimizing the design of OFET photodetectors, thereby advancing their applications in low-light detection, high-sensitivity sensing, and related fields [12,13].

## 2. Materials and Methods

Materials: Chlorobenzene (anhydrous, 99.8%), and PS (analytical standard, average molecular weight (Mw): ≈190,000) were bought from Sigma-Aldrich (St. Louis, MI, USA). C_8_-BTBT (99.9%) was purchased from Luminescence Technology Corp (New Taipei City, Taiwan, China). All materials were used directly without further purification.

Device fabrication: C_8_-BTBT and PS were blended in chlorobenzene with a weight ratio of 10:3 (mg/mL), and a glass slide was used as a substrate with the following procedures. The substrate was cleaned in detergent, deionized water, acetone, and isopropyl alcohol for 5 min separately in an ultrasonic bath and then cleaned again with acetone and isopropyl alcohol for 5 min, respectively. The substrate was dried by a 99.999% purity nitrogen flow. Subsequently, a 100 nm thick layer of aluminum was deposited on the glass substrate as a common gate electrode by thermal evaporation in a vacuum. The dielectric layer was then formed by the previous method of anodization, a constant current of 0.7 mA cm^−2^ was used, and the maximum voltage was set as 15 V; thus, the final thickness of aluminum oxide was 1.4 × 14 + 2 = 23 nm. Then, solution shearing was performed on a homemade device with an OTS-treated blade, with a shearing speed of 0.3 mm s^−1^ and 40 °C on the heat platform. Finally, F4-TCNQ and silver were successively deposited by thermal evaporation through the same shadow mask. The vacuum chamber pressure was 1 × 10^−4^ Pa, the evaporation rate of silver (30 nm in thickness) was 1 Å s^−1^, and F4-TCNQ (4 nm in thickness) was 0.5 Å s^−1^.

Characterizations: POM images were captured by a Nikon Eclipse Ci-POL-polarized microscope (Nikon, Melville, NY, USA) with a rotation stage. Out-of-plane XRD results were obtained using a Rigaku SmartLab X-ray diffractometer (Rigaku, Tokyo, Japan) with the highest power of 9 kW. SAED measurements were conducted on a Tecnai G2 F20 S-TWIN (Institut Català de Nanociència i Nanotecnologia (ICN2), Bellaterra, Spain). The transfer and output I–V curves of the field-effect transistors were measured using a Keithley 2636B digital source meter (Keithley, Cleveland, OH, USA) and probe station with dark box in air. UV light source is provided by an LED power source, and light intensity is measured by Thorlab PM100D (Thorlab, Newton, NJ, USA).

## 3. Results

**Figure 1:**
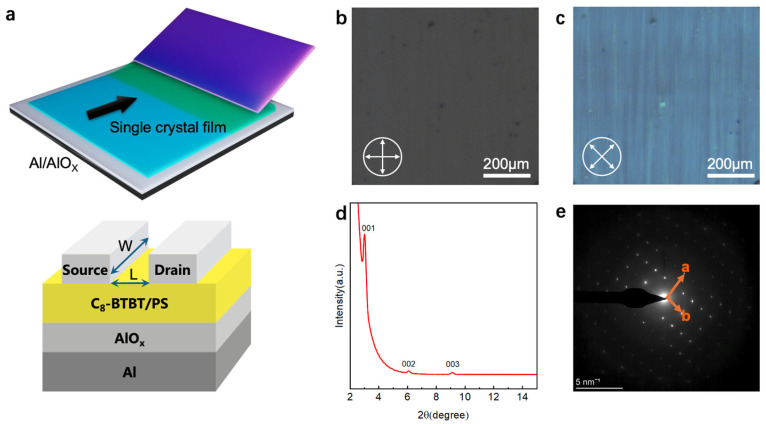
(**a**) Schematic drawing of the solution shearing method and device structure, in which channel length L and channel width W are marked with arrow. (**b**,**c**) Polarized optical microscope images of the C_8_-BTBT crystalline film obtained through the solution shearing process. (**d**) Out-plane XRD results of the C_8_-BTBT single crystals. (**e**) SAED patterns of the C_8_-BTBT single crystals.

In this study, high-performance OPTs based on single-crystal OFETs were fabricated and characterized. First, single-crystal OFETs were fabricated using the solution shearing method [1,14], which enabled precise control over crystal growth, ensuring the production of high-quality organic semiconductors with well-aligned grains. For the gate dielectric layer, anodization [5,15] was performed to construct a high-quality dielectric/semiconductor interface with a minimized density of trapped states. The source and drain electrodes were fabricated using evaporated silver in combination with an F4-TCNQ [16,17] injection layer. A schematic illustration of the solution shearing process and the device structure is presented in Figure 1a.

Following the fabrication of large-area organic single crystals through the solution shearing process, they were characterized, and the corresponding results are presented in Figure 1. Figure 1b,c depicts polarized optical microscopy (POM) images of the single crystals. These images reveal uniform light extinction when the sample is rotated by 45°, confirming the single-crystal nature of the material. Figure 1d displays the out-of-plane X-ray diffraction spectrum of the sample, where distinct diffraction peaks appear at 2θ values of 3°, 6°, and 9°, corresponding to the (001), (002), and (003) planes, respectively. The periodicity of these peaks indicates the highly ordered layered structure of the sample, further confirming its excellent crystallinity. Figure 1e presents the selected area electron diffraction pattern of the sample, which reveals a regular dot-lattice structure with clearly labeled *a*-axis and *b*-axis crystal orientations. This well-defined lattice diffraction pattern indicates high-quality single-crystal properties and confirms the periodicity of the two-dimensional crystal lattice.

Next, OFETs with varying *W*/*L* ratios and device areas were assessed under light irradiation to examine the influence of device geometry on responsivity and photocurrent generation. This approach enabled a systematic investigation into the impact of geometric configuration on the optical and electrical performance of the devices. Notably, the responsivity (*R*) of a photodetector is a key parameter that quantifies its ability to convert incident light into an electrical signal. For OFETs, responsivity is defined as the ratio of the photocurrent generated by the device to the incident optical power, which is expressed mathematically as

(1) $$R=\frac{I_{ph}}{P_{opt}}$$

where *I*_ph_ denotes the photocurrent, measured in amperes (A); and *P*_opt_ represents the incident optical power, measured in watts (W). Specifically, *P*_opt_ is defined as the product of the incident light intensity and the active region area.

**Figure 2:**
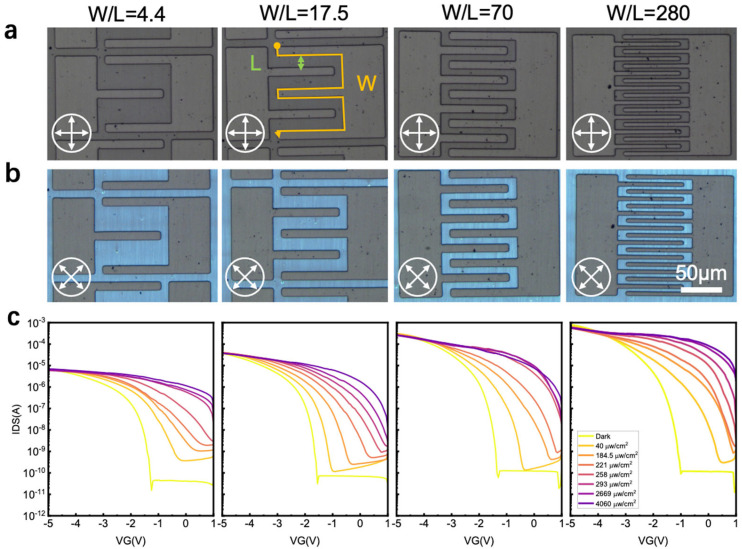
(**a**,**b**) Polarized optical microscope images of the same area C_8_-BTBT OFET devices. (**c**) Transfer I-V curves under various UV light intensities, ranging from 40 μW/cm^2^ to 4060 μW/cm^2^, as well as in the dark.

First, the *W*/*L* ratios of the OFETs were varied while maintaining their channel area constant (Figure 2). The results revealed that the incident optical power remained unchanged for all devices under identical light intensities. During this test, a 365 nm light-emitting diode was used as the light source. Figure 2a,b presents POM images of the OFETs with progressively increasing *W*/*L* ratios (4.4, 17.5, 70, and 280). Despite the geometric variations, the uniformity and clarity in the POM images indicate high-quality fabrication of the crystals and well-aligned molecular arrangements, which facilitate efficient charge transport along the channels. Notably, the photosensitive area for all of the devices was fixed at 0.00448 cm^2^, ensuring consistent light absorption across all samples regardless of the *W*/*L* ratio or overall device dimensions.

The transfer curves in Figure 2c illustrate the *I*_DS_–*V*_G_ characteristics of the devices under varying ultraviolet light intensities, ranging from dark conditions to a maximum value of 4060 µW/cm^2^. For each device, the transfer *I*–*V* curves shift to the positive direction under light irradiation, demonstrating typical photoresponse behavior. Details regarding the operating mechanism of phototransistors can be found in our recent publication [18] and briefly explained as follows. When applying a positive gate bias to the device under UV light illumination, the mobile electrons generated by the photon in the organic semiconductor would be captured by the PS electret. The electron traps positively shift the Vth (this can be considered as helping the formation of the transistor channel at a lower gate voltage). As a result, the *I*_DS_ will increase. The number of charges being trapped can be further controlled by tuning the incident light intensity at a particular writing bias. Across different devices, as the *W*/*L* ratio increases, both the dark current and the photocurrent (*I*_ph_) increase considerably. Notably, given that the active area of all OFETs is maintained to be constant, the incident optical power remains identical across devices. Because the channel current in OFETs is proportional to the *W*/*L* ratio, devices with a larger *W*/*L* ratio more efficiently convert photon energy into electrical output. Consequently, the overall photocurrent and photoresponse can be modulated by adjusting the *W*/*L* ratio under identical lighting conditions.

**Figure 3:**
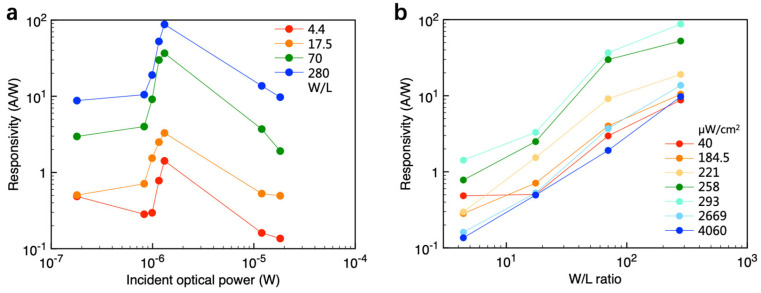
(**a**) Relationship between responsivity and incident optical power for various W/L ratios. (**b**) Relationship between responsivity and the W/L ratio for various incident light intensities.

The responsivity data of the devices as a function of incident optical power and W/L ratio were extracted from Figure 2c and are plotted in Figure 3a and Figure 3b, respectively. As the incident optical power increases from 10^−7^ W to 2 × 10^−6^ W, the responsivity rises rapidly. This increase occurs because photoinduced electrons become trapped in defect states within the polymer electret or semiconductor, leading to a positive shift in the threshold voltage. Consequently, the drain–source current and the photocurrent (I_ph_) increase by several orders of magnitude owing to gate modulation in the FET, resulting in a substantial enhancement in responsivity. As the incident optical power further increases to approximately 10^−5^ W, corresponding to a light intensity above 2000 µW cm^−2^, the number of trapped photoinduced electrons tends to saturate. Correspondingly, as illustrated in Figure 2c, the transfer *I*–*V* curves of most devices exhibit minimal shifts at incident light intensities above 2000 µW cm^−2^. The increment of output current is smaller than the increment of incident optical power. This implies that responsivity decreases with increasing light intensity.

Unlike the incident optical power, responsivity exhibits a continuous enhancement with an increasing *W*/*L* ratio. The log–log scale plot in Figure 3b reveals a strong linear relationship between responsivity and the *W*/*L* ratio for most of the data except for the device under 40 μW cm^−2^ illumination. This is probably due to the device-to-device variation. Although an organic single crystal is used for device fabrication, device uniformity can be further improved. Interface trap states or contact resistance variation would lead to the measurement results deviating from the linear relationship. These results suggest that without changing the incident light intensity, the responsivity of a phototransistor can be increased by nearly two orders of magnitude by tuning the *W*/*L* ratio within a reasonable range.

This geometric effect on responsivity may arise from its fundamental definition. In diode-like photodetectors, such as photodiodes, responsivity is independent of device geometry because both the output current and the incident optical power are normalized by the device area. Specifically, in a photodiode, the output current is given by the product of the current density and the device area, while the incident optical power is the product of the incident light intensity and the device area. Consequently, the device area cancels out when calculating responsivity. However, phototransistors behave differently. In transistor-based devices, the output current is proportional to the *W*/*L* ratio rather than the device area. Notably, the same device area can correspond to different channel widths and lengths, as illustrated in Figure 2. Given that the output current in transistors is modulated by the *W*/*L* ratio, responsivity exhibits an evident dependence on geometry. Therefore, increasing the *W*/*L* ratio offers a straightforward approach to effectively enhancing the responsivity of OPTs.

**Figure 4:**
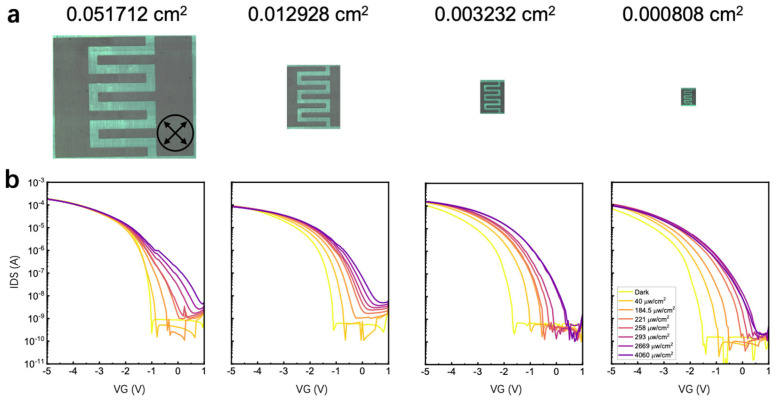
(**a**) Polarized optical microscope images of devices with four different areas: 0.051712 cm^2^, 0.012928 cm^2^, 0.003232 cm^2^, and 0.000808 cm^2^, (**b**) The corresponding transfer I–V curves under various UV light intensities, ranging from 40 μW/cm^2^ to 4060 μW/cm^2^, as well as in the dark.

After experimentally varying the *W*/*L* ratio and output current, we further examined the geometric effect on responsivity by altering the overall device area. During this analysis, the *W*/*L* ratio for all four devices was maintained constant at 32, while both *W* and *L* were proportionally reduced. As depicted in Figure 4a, the active region area shrank by a factor of 64, implying that under the same incident light conditions, the incident optical power used in responsivity calculations was also reduced by a factor of 64. The POM images in Figure 4a confirm the uniformity of the devices with varying areas. Despite the reduction in area, the devices maintain consistent geometric configurations and exhibit high crystal quality.

Figure 4b depicts the transfer *I*–*V* characteristics of the devices under varying illumination intensities. Given that the *W*/*L* ratio remains constant, the drain–source current in the dark is nearly identical for all devices. Notably, reducing the device area does not result in a proportional decrease in photocurrent. However, the photocurrent increases slightly in devices with shorter channel lengths. This likely occurs because the threshold voltage shift induced by photo-generated trapped charges intensifies at shorter channel lengths owing to the drain-induced barrier lowering effect [19].

**Figure 5:**
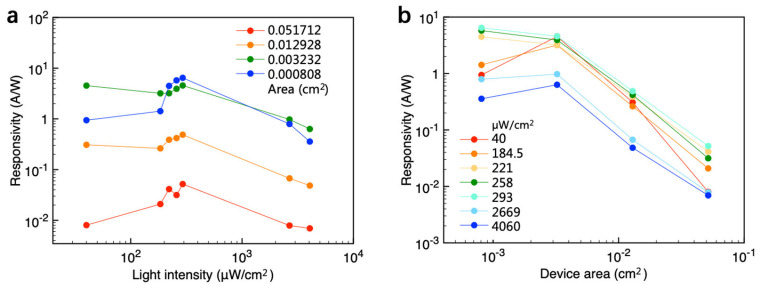
(**a**) Relationship between responsivity and incident light intensity for various device areas. (**b**) Relationship between responsivity and device area for various incident light intensity.

Similarly, the responsivity data of the devices under varying incident light intensities and device areas were extracted from Figure 4b and are plotted in Figure 5a and Figure 5b, respectively. As the incident light intensity increases from 40 µW cm^−2^ to below 300 µW cm^−2^, the responsivity generally increases due to a photo-induced positive shift in the threshold voltage. However, when the incident light intensity exceeds 2000 µW cm^−2^, the number of photo-induced trapped electrons tends to saturate. Consequently, the transfer *I*–*V* curves exhibit minimal shift, and the responsivity decreases at higher light intensities.

Figure 5b plots the responsivity as a function of device area, revealing a clear linear trend until the device area decreases below 0.003232 cm^2^. This strong linearity is attributed to the definition of responsivity. Specifically, varying the device area under a fixed light intensity leads to a proportional change in incident optical power. Consequently, if the output current remains unchanged, the responsivity increases as the device area decreases. However, reducing the device area further leads to responsivity saturation, indicating that device performance is affected at shorter channel lengths. For instance, at shorter channel lengths, contact resistance becomes the primary performance-determining factor in C_8_-BTBT OFETs owing to the low-lying highest occupied molecular orbital energy level (5.6–5.8 eV) of the C_8_-BTBT semiconductor. The results in Figure 5b indicate that simply reducing the active region area, without altering the device structure, can enhance responsivity by more than two orders of magnitude.

These results, combined with the findings in Figure 3b, indicate the effect of device geometry on the responsivity of OPT devices. Specifically, the results suggest that adjusting the channel width and channel length of the phototransistor, without altering the incident light intensity or device structure, can enhance responsivity by nearly 100 times. This substantial improvement in responsivity arises from the fact that, unlike in photodiodes, output current in phototransistors is modulated by both the channel dimensions and the threshold voltage shift, rather than being strictly proportional to incident optical power. The channel dimensions not only directly influence the output current but also determine the active region area, which is incorporated into responsivity calculations. To achieve higher responsivity, shorter-channel-length devices are preferred. This is because a device with a shorter channel length enables an increase in its *W*/*L* ratio without expanding the active region area or allows the *W*/*L* ratio to remain constant while reducing the active region area, both of which lead to enhanced responsivity. However, as the channel length decreases, contact resistance becomes a critical limiting factor in organic transistors. Generally, maintaining efficient charge injection at reduced channel lengths requires low contact resistance. The contact resistance of OFETs is relatively larger compared to FETs based on other semiconductors such as transition metal dichalcogenides (TMDs) [20] and carbon nanotubes [21]. Consequently, the relatively high contact resistance of OFETs hinders further improvements in responsivity through channel length reduction.

## 4. Discussion

In a phototransistor, excitons are generated under incident light irradiation, following which they dissociate under the influence of the gate field. The electrons or holes resulting from this dissociation can become trapped either at the semiconductor/dielectric interface or within the semiconductor itself. This trapping alters the local electric field in the channel region, leading to a shift in the threshold voltage (*V*th) and a corresponding increase in output current. The shift in *V*th directly reflects the variation in trapped charge density, which is defined as follows:

(2) $$∆n=\frac{∆V_{th}C_{i}}{q}$$

where *C*_i_ denotes the unit-area capacitance, *q* represents the elementary charge, and *Δn* denotes the trapped charge density (cm^−2^). If the exciton generation rate, exciton dissociation rate, and charge trapping efficiency remain constant in a given device, the trapped charge density becomes proportional to the incident light intensity until saturation. This suggests that *ΔV*_th_ is primarily determined by incident light intensity and remains independent of device area. Thus, given that *ΔV*_th_ remains unaffected by device area under a fixed light intensity, the increase in photocurrent (*I*_ph_) caused by *ΔV*_th_ is also independent of area. Considering the definition of responsivity, *R* = *I*_ph_/*P*_opt_, where *P*_opt_ is defined as the product of incident light intensity and the active region area, responsivity (*R*) should be inversely proportional to the active region area when all other parameters remain constant. The observed linear relationship between *R* and device area in Figure 5b successfully verifies this statement.

Furthermore, the drain–source current of a phototransistor under illumination is given as *I*_DS_ = *I*_ph_ + *I*_dark_, which is proportional to the *W*/*L* ratio, and because *I*_DS_ ≈ *I*_ph_, responsivity *R* is also proportional to the *W*/*L* ratio at a fixed *P*_opt_, as evidenced by the results in Figure 3b. To accurately evaluate device performance, we propose the use of a channel-width-normalized responsivity (*R*′) to characterize OPTs. The normalized responsivity is defined as

(3) $$R‘=\frac{R}{W}=\frac{I_{ph}/W}{P_{opt}}$$

Here, by dividing the photocurrent by *W*, the contribution of channel width to *I*_ph_ is normalized, enabling a geometry-independent evaluation of device performance. This correction allows for a direct comparison across devices with varying *W*/*L* ratios or total areas.

**Figure 6:**
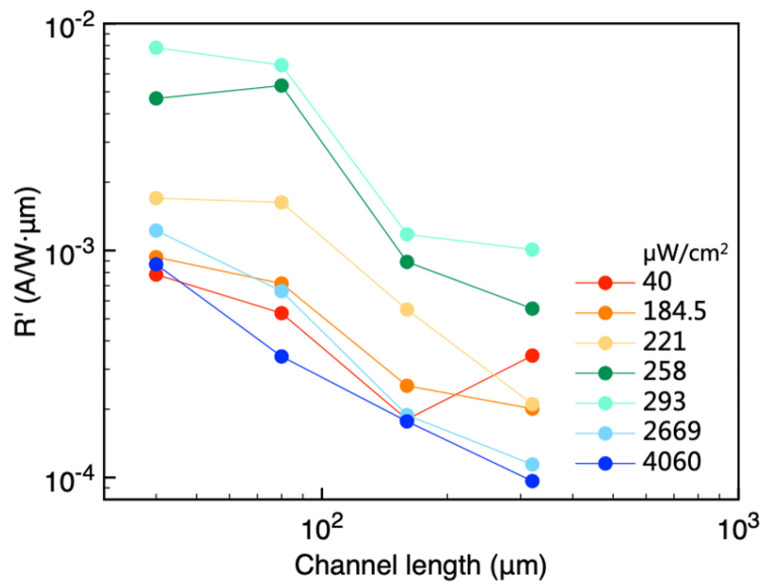
Width normalized responsivity as a function of channel length.

We applied this normalization to the data in Figure 3b and plotted *R*′ as a function of channel length (Figure 6). Even without multiplying by channel width, responsivity still exhibits a strong dependence on channel length. The data in Figure 6 confirm the conclusion from Section 3 that devices with shorter channel lengths generate higher output currents. However, channel length reduction effectively enhances device performance until contact resistance becomes a limiting factor.

## 5. Conclusions

In summary, this study systematically investigated the effect of OPT geometry on responsivity. Our results revealed that for a fixed device area and incident light intensity, responsivity is proportional to the *W*/*L* ratio, whereas for a fixed *W*/*L* ratio, responsivity is inversely proportional to the device area. This dependence of responsivity on device geometry arises because the output current in phototransistors is not strictly proportional to incident optical power but is also influenced by the channel dimensions and threshold voltage shift. To address these geometric effects, we introduced a channel-width-normalized responsivity parameter, allowing for a more accurate evaluation of device performance across different device structures. Overall, this study provides valuable insights into the characteristics of OPTs and highlights the potential for device optimization through geometric parameter tuning.

## Data Availability

The original contributions presented in this study are included in the article. Further inquiries can be directed to the corresponding author.
